# Feasibility and pitfalls of combining small subpopulations for genome‐wide association analysis of maize (*Zea mays* L.) kernel traits

**DOI:** 10.1002/tpg2.70319

**Published:** 2026-10-08

**Authors:** Myeong‐Geon Seok, Seongmin Hong, Jiyun Go, Jin Seong Park, Hobin Lee, Sanghak Lee, Gibum Yi

**Affiliations:** ^1^ Department of Bio‐Environmental Chemistry, College of Agriculture and Life Sciences Chungnam National University Daejeon South Korea

## Abstract

Genome‐wide association studies (GWASs) typically require large populations to achieve sufficient statistical power; however, maintaining large populations is labor‐intensive and costly. In this study, we evaluated the feasibility of improving GWAS detection power by strategically integrating small maize (*Zea mays* L.) subpopulations for kernel‐related traits. Using high‐throughput phenotyping of eight kernel traits, GWAS was conducted across four different population combinations. Expanding population size generally increased single‐nucleotide polymorphism (SNP) association strength and enabled identification of additional loci, supporting the advantage of subpopulation integration. However, some loci identified in smaller combinations lost association strength after incorporating genetically divergent subpopulations, indicating that phenotypic distribution and genetic background strongly influence GWAS outcomes. Comparison with previously reported kernel quantitative trait loci revealed that many identified SNPs reside in multi‐trait genomic regions, while several loci exhibited trait‐specific effects. Candidate genes within linkage disequilibrium blocks included pentatricopeptide repeat proteins, an insulin‐degrading enzyme‐like protein, and a peptidyl‐prolyl cis–trans isomerase, all with plausible roles in kernel development. Collectively, our results suggest that integrating small populations is an effective and practical strategy to enhance GWAS feasibility and locus discovery, while careful consideration of population structure remains essential.

## Core Ideas

- Integrated maize subpopulations were used to evaluate the feasibility of combining small‐scale subpopulations for genome‐wide association studies (GWASs).
- Combining subpopulations increased the statistical power to detect associated loci while revealing the benefits and limitations of this strategy for GWAS.
- Several single‐nucleotide polymorphisms (SNPs) identified across population combinations overlapped with previously reported quantitative trait loci regions.
- Based on these SNPs, several candidate genes within linkage disequilibrium blocks were identified as potential regulators of kernel development.

## Plain Language Summary

To develop better crops, scientists need to find the specific genes responsible for important traits, like the size or quality of maize kernels. Usually, this requires growing and studying thousands of plants, which is very expensive and time‐consuming. In this study, we tested a more efficient approach: instead of creating one massive group of plants, we strategically combined several smaller, existing groups of maize. Our results showed that combining these smaller groups generally made it easier to pinpoint important genetic locations. However, we also found that if the groups being combined are too different from each other, it can sometimes hide certain genetic signals. We successfully identified several genes that likely play key roles in how maize kernels develop. This research proves that researchers can save time and money by smartly grouping smaller plant populations together to discover important genes, as long as they carefully account for the natural differences between those groups.

**Abbreviations**
CD: centroid deviation
GWAS: genome‐wide association study
LD: linkage disequilibrium
LWR: length‐to‐width ratio
QTL: quantitative trait loci
SNP: single‐nucleotide polymorphism

## 1 INTRODUCTION

Maize (*Zea mays* L.) is one of the world's major staple crops along with rice (*Oryza sativa* L.) and wheat (*Triticum aestivum* L.) (Awika, 2011). It is utilized as food and animal feed as well as raw materials for industrial resources such as biofuels and biodegradable products (Ranum et al., 2014). Maize kernels are final products of cultivation, directly associated with crop yield. They preserve and transmit diverse genetic information from their parents to next generations as seeds (Waters et al., 2011). Therefore, understanding the genetic factors that regulate kernel‐related traits is essential for improving yield and securing desirable genetic variations for breeding. In this context, previous studies profiled key genes and genetic factors involved in maize kernel development and kernel‐related traits, including endosperm development, starch accumulation, storage reserve deposition, and kernel morphology (reviewed in Dai et al., 2021).

As genomic resources and infrastructure—including public databases, high‐quality reference genomes, and high‐throughput genotyping platforms—have rapidly advanced and become widely accessible, numerous studies have identified useful genetic factors through genome‐wide analyses (Land et al., 2015; Morrell et al., 2012; Zhernakova et al., 2024). Although genome‐wide association study (GWAS) is a powerful tool for identifying genetic variations associated with specific traits (Uffelmann et al., 2021), certain limitations persist, particularly in plant studies. Notably, GWAS tends to reduce sensitivity for variants with small effect sizes due to its emphasis on detecting a limited set of core loci, and it constrains the discovery of rare variants that may be infrequent in the population (Park et al., 2010).

The power of GWAS also depends on the accuracy and consistency of phenotypic measurements, which can be substantially influenced by environmental variation. In this regard, kernel‐related traits such as kernel length, width, thickness, and weight have been reported to show high broad‐sense heritability (*H* ^2^), often exceeding 80%, across previous studies (M. Johnson et al., 2019; Millet et al., 2019). This suggests that phenotypic variation in these traits is predominantly determined by genetic factors rather than environmental influences, making them particularly suitable for GWAS‐based identification of loci associated with kernel development (J. Liu et al., 2017; M. Liu et al., 2020; Wen et al., 2014).

Additionally, utilizing both GWAS and quantitative trait loci (QTLs) mapping for identifying specific genomic regions associated with phenotypic variation is an effective strategy to pinpoint causal genes (J. Chen et al., 2016; Gutierrez & Torres, 2025; J. Liu et al., 2017; Pang et al., 2020; Tao et al., 2020). By identifying overlapping genomic regions consistently identified across different analytical methods, researchers can pinpoint the most robust and reliable loci associated with target traits while simultaneously enhancing statistical power and mapping resolution. Regarding this integration, Qi et al. (2025) reported that expanding flanking regions between GWAS‐detected quantitative trait genes and QTLs increases the probability of identifying causal genes. This strategy has also been widely reported in studies of crop yield (W. Chen et al., 2016; Gutierrez & Torres, 2025; Tao et al., 2020).

For ensuring adequate detection, a substantial number of inbred lines is required for GWAS. A previous study recommended using a sample size of more than 100 accessions for plant GWAS and reported that increasing the sample size to 192 accessions improved statistical power by more than twofold (Atwell et al., 2010). Additionally, GWAS simulations of wheat heading date demonstrated that progressively increasing sample size from 100 to 200 individuals showed the most dramatic gains in QTL detection power, with further improvements up to 300–500 before plateauing, while also enhancing the accuracy of marker effect estimates (Lell et al., 2024). Therefore, to improve GWAS statistical power, it is necessary to establish a substantial population consisting of diverse inbred lines.

However, inbreeding depression often leads to a decline in seed production and plant vigor over successive generations of self‐pollination (Han et al., 2021; Yan et al., 2017). Additionally, ensuring spatial isolation to prevent cross‐pollination requires specialized facilities (L. Zhang et al., 2020). Therefore, maintaining and cultivating large‐scale populations consisting of inbred lines is labor‐intensive, costly, and time‐consuming, thereby limiting the feasibility of GWAS in small‐scale laboratory settings.

To overcome these limitations, integrating subpopulations into GWAS analysis may provide a useful approach. Combining genetically distinct maize populations can increase statistical power and broaden the range of segregating alleles, potentially facilitating the detection of additional candidate loci, although substantial population differentiation may also introduce confounding effects (Hamazaki et al., 2020; Zuffo et al., 2022). Therefore, genetic structure, allele‐frequency distribution, phenotypic compatibility, and the extent to which population structure and relatedness can be adequately controlled. Additionally, GWAS conducted in distinct subpopulations may detect only alleles segregating within those specific groups. Therefore, comparing whole‐population and subpopulation‐level analyses can help identify subpopulation‐specific haplotypes (Skovbjerg et al., 2025). However, there have been limited studies investigating the effects of integrating small‐scale subpopulations into GWAS population. This strategy could be highly effective for utilizing small subpopulations developed in small‐scale laboratory settings, or subgroups that may possess unique and rare genetic variations but are otherwise too small to provide sufficient power for independent GWAS. By integrating small‐scale subpopulations into other subpopulation, researchers can not only enhance statistical power by increasing the overall population size but also expect the identification of novel candidate loci driven by the effects of added subpopulations.

In this study, we evaluated the validity of integrating small subpopulations to increase the statistical power of GWAS. We compared GWAS results across four different subpopulation combinations and observed enhanced strength of the signal and the identification of additional candidate loci as the subpopulations were combined. Moreover, by identifying common single‐nucleotide polymorphisms (SNPs) that overlapped with previously reported maize kernel‐related QTL regions, we selected D for further analysis. Finally, phenotypic distributions among haplotypes of these prioritized SNPs are significantly distinct; thus, we confirmed that our approach was a valid and highly effective strategy in GWAS. These prioritized SNPs provide promising targets for candidate gene discovery and may facilitate the genetic improvement of maize kernel‐related traits.

**Core Ideas**

- Integrated maize subpopulations were used to evaluate the feasibility of combining small‐scale subpopulations for genome‐wide association studies (GWASs).
- Combining subpopulations increased the statistical power to detect associated loci while revealing the benefits and limitations of this strategy for GWAS.
- Several single‐nucleotide polymorphisms (SNPs) identified across population combinations overlapped with previously reported quantitative trait loci regions.
- Based on these SNPs, several candidate genes within linkage disequilibrium blocks were identified as potential regulators of kernel development.

## 2 MATERIALS AND METHODS

### 2.1 Plant materials

A total of 168 maize inbred lines from three distinct subpopulations were used in this study. These subpopulations consisted of 93 accessions from the Rural Development Administration, Korea (hereafter RDA; Son et al., 2025), 26 maize nested association mapping founders, including B73 (hereafter NAM; Flint‐Garcia et al., 2005), and 49 Korean maize landraces (hereafter CNU; Hong et al., 2024). Maize kernels used in this study were obtained from seeds harvested from plants grown at the CNU experimental farm during either the 2023 or 2024 growing season, depending on seed availability. The germplasm resources used in this study, identified by their accession numbers (prefixed with “IT”) in Table S1, were obtained from the national agrobiodiversity center of the RDA, Republic of Korea. Procedures for DNA extraction and library construction for the Illumina NovaSeq 6000 platform were performed as described previously (Park et al., 2025).

### 2.2 Measurement of kernel phenotypes

To perform high‐throughput phenotyping of the collected kernels, *SmartGrain 1*, an image‐based software program described by Tanabata et al. (2012), was used to measure grain size traits, including kernel width, kernel length, projected kernel area size (AS), and kernel perimeter length (PR), as well as shape‐related traits such as kernel circularity (CS), length‐to‐width ratio (LWR), and centroid deviation (CD, distance between intersection of length and width and center of gravity) (Figure S1A,B). To validate measurement accuracy, 10 accessions were randomly selected from the 168 accessions, and 10 kernels per accession (100 kernels in total) were measured. Kernel length and width obtained from *SmartGrain 1* were compared with manual measurements using Vernier calipers by Pearson's correlation analysis, as these traits can be directly measured by both methods. The validation results are shown in Figure S1C,D. Kernel thickness was additionally measured using digital Vernier calipers (SYATEK, Stainless Steel Vernier Caliper J50). Phenotypic data were recorded from 10 kernels per accession to characterize kernel variation. The mean value for each trait was calculated and used for subsequent GWAS analysis.

### 2.3 Generation of genotype data of maize populations

The RDA panel was genotyped using the Maize600k SNP array (Son et al., 2025). For the NAM founders, including B73, Illumina paired‐end resequencing data were obtained from the NCBI Sequence Read Archive under BioProject IDs including PRJEB31061, PRJEB32225, and PRJEB32404. The CNU panel was available under BioProject PRJNA1222583 (Table S1). Quality filtering of raw reads, genotype calling, and the integration of chip genotype data with variant call format (VCF) files were performed following the methods described in Son et al. (2025).

To assess the effect of genetic diversity on GWAS performance, subpopulations were constructed by combining the RDA, NAM, and CNU panels in different combinations. Four subpopulations were generated: RDA (93 accessions), RDA + NAM (119 accessions), RDA + CNU (142 accessions), and RDA + NAM + CNU (168 accessions). VCF files were prepared for each of the four population combinations for downstream analyses. Within each population combination, variant sites were filtered using a minor allele frequency threshold of >0.05 and a missing genotype rate of <0.05. Among the SNPs that passed these filtering criteria, 537,612 SNPs shared across all four population combinations were retained for GWAS and the corresponding multiple‐testing correction, enabling consistent comparison across population combinations.

### 2.4 Linkage disequilibrium pruning and population structure analysis

To characterize genetic diversity and population structure, and to assess shifts in genetic architecture associated with the incremental addition of independent panels, principal component analysis (PCA) was performed using PLINK v1.9 (Chang et al., 2015; Purcell et al., 2007). The proportion of variance explained by each principal component was calculated from corresponding eigenvalues. Subsequently, genome‐wide linkage disequilibrium (LD) decay was estimated separately for each panel using PopLDdecay (C. Zhang et al., 2019).

To reduce redundancy among tightly linked SNPs, LD‐based SNP pruning was performed using PLINK v1.9 with the following parameters: a sliding window of 50 SNPs, a step size of 10 SNPs, and an *r* ^2^ threshold of 0.4. The resulting LD‐pruned SNP dataset contained 103,664 SNPs and was used for both ADMIXTURE‐based population structure analysis and IQ‐TREE‐based phylogenetic analysis (Alexander et al., 2009). The number of ancestral populations (*K*) was tested from 1 to 20 with 10 replicates for each *K* to determine the optimal number of subgroups among the 168 maize accessions in this study. Furthermore, to investigate the genetic relationships among accessions belonging to different panels, a maximum‐likelihood phylogenetic tree for the 168 accessions was constructed using IQ‐TREE v2 (Minh et al., 2020) with 1000 bootstrap replicates for evaluation of the node support.

### 2.5 GWAS and identification of candidate genes

To identify SNPs associated with kernel traits, GWAS was conducted using the fixed and random model circulating probability unification (FarmCPU), Bayesian‐information and linkage‐disequilibrium iteratively nested keyway (BLINK), and mixed linear model (MLM) models implemented in genome association and prediction integrated tool (GAPIT) (Hoffman, 2013; Lipka et al., 2012; Z. Zhang et al., 2010; J. Wang & Zhang, 2021). ADMIXTURE analysis was performed independently for each population combination, including RDA, RDA + NAM, RDA + CNU, and the whole population. For each panel, the optimal *K* value was determined based on the cross‐validation (CV) error, and the corresponding Q matrix inferred from that specific population combination was included as a fixed‐effect covariate to control population structure. A suggestive threshold was defined as −log_10_(1/*N*), where *N* represents the total number of SNPs used in the analysis (537,612 SNPs; −log_10_ (*p*) = 5.73). Previous studies have suggested that Bonferroni correction may be overly conservative in GWAS because SNPs are not fully independent due to LD, whereas false discovery rate (FDR)‐ and SimpleM‐based approaches can provide useful alternatives for multiple‐testing correction by accounting for FDRs or the effective number of independent markers (R. C. Johnson et al., 2010; Tibbs‐Cortes et al., 2021). Following Gao et al. (2008), the effective number of independent tests was estimated using an experiment‐wise type I error rate of *α_e_* = 0.05 and a cumulative variance cutoff of *C* = 0.995. The 537,612 SNPs were divided into chromosome‐specific blocks of up to 150 consecutive markers, and block‐wise effective test numbers were estimated from eigenvalue decomposition of SNP correlation matrices and summed to obtain the genome‐wide effective number of independent tests (*M*_eff,G_). The resulting *M*_eff,G_ values were 103,655, 136,726, 146,826, and 167,430 for RDA, RDA + NAM, RDA + CNU, and RDA + CNU + NAM, respectively. SimpleM significance thresholds were calculated as *α_e_*/*M*_eff,G_, and the corresponding SimpleM‐ and Benjamini–Hochberg FDR‐based thresholds are summarized in Table S2. However, to maintain consistency in SNP selection and facilitate direct comparison across traits and population combinations, the suggestive threshold was applied as the primary criterion for identifying SNPs in the GWAS results and the SimpleM‐derived significance threshold was used to select prioritized SNPs for further analysis (Table S2). Candidate SNPs identified in each panel were annotated using SnpEff (Cingolani et al., 2012) based on the B73 reference genome (AGPv2) annotation (Schnable et al., 2009). To map AGPv2 gene models to the current Zm‐B73‐REFERENCE‐NAM‐5.0 gene set (Hufford et al., 2021), protein similarity searches (all‐versus‐all BLASTP‐like comparisons) were performed using DIAMOND (Buchfink et al., 2015).

### 2.6 QTL overlap, LD block characterization, and single‐marker association analysis

Published QTLs were included only if they were associated with maize kernel‐related traits and provided sufficient marker information, such as flanking or peak markers, to enable the assignment of genomic coordinates. A conservative 0.5‐Mb window was applied around the reported QTL regions to identify SNPs associated with kernel size. LD blocks were characterized using LDBlockShow (Dong et al., 2021). Single‐marker genotype–phenotype association was conducted for the eight traits. Statistical analyses were performed in R v4.3.2 using a custom pipeline. Genotype groups with fewer than two individuals were excluded from the analysis. Welch's *t*‐test was employed for comparisons between two groups. For comparisons involving three or more groups, analysis of variance with Type III Sum of Squares was performed to account for unequal group sizes. Pairwise post hoc comparisons were adjusted using the Benjamini–Hochberg (BH) method, and statistical significance for these comparisons was determined based on BH‐adjusted *p*‐values < 0.05.

## 3 RESULTS

### 3.1 Kernel traits differ significantly across subpopulations and kernel types

The three panels—RDA (*n* = 93), CNU (*n* = 49), and NAM (*n* = 26)—comprised a total of 168 maize accessions. Eight kernel morphology‐related traits were measured for all accessions (Figure 1A–H; Table S3). Based on kernel type, the whole panel was classified into flint (*n* = 71), waxy (*n* = 49), dent (*n* = 29), sweet (*n* = 18), and popcorn (*n* = 1) (Figure 1I; Table S4).

**FIGURE 1:**
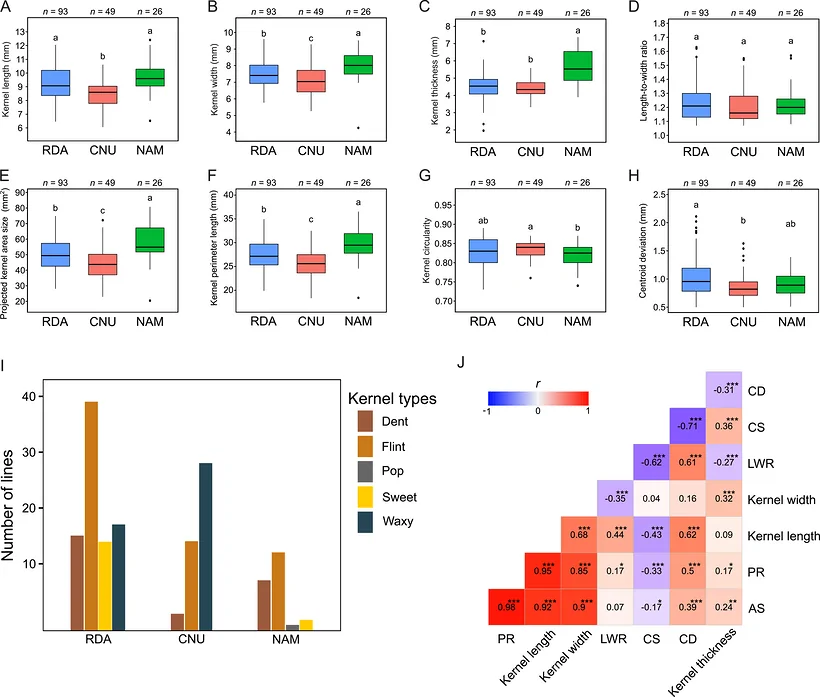
Phenotypic distribution of three subpopulations on eight kernel‐related traits (kernel length, kernel width, kernel thickness, length‐to‐width ratio [LWR], projected kernel area size [AS], kernel perimeter length [PR], kernel circularity [CS], centroid deviation [CD]). The RDA (Rural Development Administration), CNU (Chungnam National University), and NAM (Nested Association Mapping) subpopulations consist of 93, 49, and 26 accessions, respectively. (A–H) Eight kernel‐related traits of three subpopulations. Seven kernel traits were measured by grain analysis program, *SmartGrain 1* and thickness was measured by digital Vernier calipers. Statistically significant differences (*p* < 0.05) among subpopulations are denoted by distinct letters above the boxes, as determined by Duncan's multiple range test (DMRT). (I) Kernel type composition of the three subpopulations. (J) Pearson's correlation matrix for the eight kernel‐related traits. The color intensity reflects the magnitude of the relationship between variables. Both the color intensity and the numerical values and asterisks within each cell indicate the direction and strength of the correlation (*r*) and their significance level, where red and blue denote positive and negative correlations, respectively.

Phenotypic correlation analysis revealed strong positive correlations among kernel size‐related traits. AS, PR, kernel length, and kernel width showed very strong positive correlations (0.85–0.98), whereas kernel length and width were moderately correlated (0.68). Among shape‐related traits, LWR–CD (0.61) and CS–CD (−0.71) exhibited moderate correlations. Moderate positive correlations were also observed between size and shape traits, including kernel length–LWR (0.44), kernel length–CD (0.62), and PR–CD (0.5). In contrast, kernel thickness showed weak correlations with the other seven traits, suggesting that it may be regulated largely independently (Figure 1J and Figure S2).

Comparison of the three subpopulations showed no significant differences for LWR. However, AS, PR, kernel length, and kernel width generally followed the trend NAM ≥ RDA > CNU, while kernel thickness exhibited the pattern NAM > RDA ≈ CNU. These results indicate that NAM founders tend to have larger kernels compared with the other panels. When stratified by kernel type, waxy maize displayed the highest kernel circularity (CS) (Figure S3O; Table S4). The relatively high proportion of waxy lines within the CNU panel likely contributes to its elevated mean CS compared with the other panels (Figure 1G,I; Table S4).

### 3.2 Population structure reflects kernel type and genetic background

The extent of LD was evaluated across the whole panel of 168 maize inbred lines using approximately 600K SNP markers. Although the overall LD decay patterns were similar among the three subpopulations, LD decayed most rapidly with increasing genomic distance in the CNU population, followed by the NAM and RDA populations. (Figure S4A). The PCA revealed three major clusters largely corresponding to kernel type (Figure 2A). Sweet maize lines (*n* = 18) formed the most genetically distinct and isolated group. Waxy maize (*n* = 49) constituted a second well‐defined cluster. In contrast, normal maize (flint and dent; *n* = 100) was grouped together but displayed internal substructure reflecting their group origins. The single popcorn accession (*n* = 1) was located between the waxy and normal maize clusters. Similarly, LD decay patterns were notably more distinct among kernel types than among subpopulations (Figure S4B).

**FIGURE 2:**
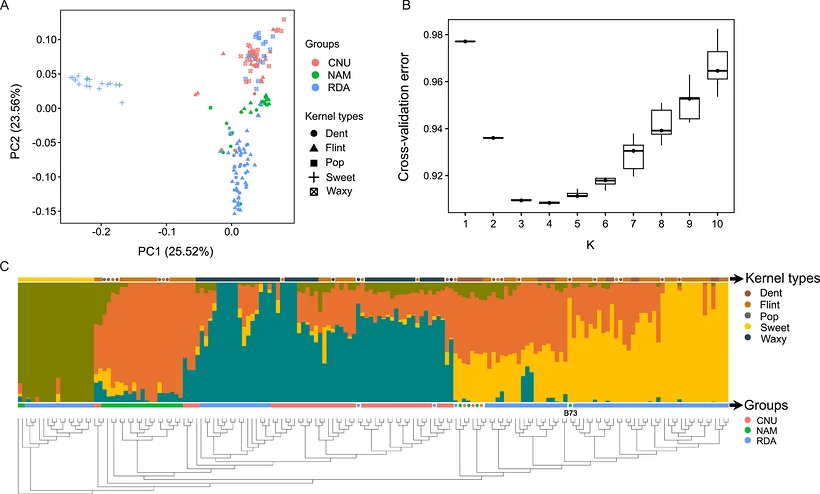
Population structure of 168 maize inbred lines combined with three subpopulations. (A) Principal component analysis (PCA) showing genetic differentiation among 168 lines based on selected 103,664 single‐nucleotide polymorphisms (SNPs). Points are colored by subpopulation: Rural Development Administration (RDA) (blue), Chungnam National University (CNU) (red), and Nested Association Mapping (NAM) (green), with point shapes indicating their kernel types. (B) Cross‐validation (CV) error plot from ADMIXTURE analysis. *K* represents the number of assumed ancestral populations (*K* = 1–10). For each *K*, 10 independent replicates were performed to estimate the CV error. (C) Population structure of 168 accessions at *K* = 4 integrated with their phylogenetic relationship. The ADMIXTURE bar plot is aligned with the topology of the maximum‐likelihood phylogenetic tree. Each vertical bar represents an accession, and colored segments indicate the proportional contribution of the four ancestral populations. The colored horizontal bar and dots above and below the plot indicate the subpopulation assignment and kernel type of each line, respectively.

Population stratification was further assessed using ADMIXTURE. CV analysis indicated the lowest CV error at *K* = 4 (Figure 2B). However, the difference in CV error between *K* = 3 and *K* = 4 was minimal. Therefore, we compared the ADMIXTURE plots generated under both *K* values. The *K* = 4 model resolved an additional genetic cluster that was not detected under *K* = 3 (Figure S4C). Furthermore, for the RDA + NAM panel, the CV error was lowest at *K* = 4, unlike the other population combinations (Figure S4D–F). These results suggest that *K* = 4 more effectively captured the underlying population structure, particularly the distinct genetic subgroup within the NAM founders. Therefore, *K* = 4 was selected for the final ADMIXTURE analysis.

The ADMIXTURE plot largely mirrored the PCA results, with clustering driven primarily by kernel type rather than group origin. All sweet maize lines were assigned predominantly to one ancestry group, whereas most waxy lines formed another distinct group. RDA and NAM lines were distributed across multiple ancestry components, reflecting their broader kernel‐type composition, whereas CNU lines were largely confined to the waxy cluster.

Notably, one additional ancestry group (*n* = 21) formed a distinct cluster independently, regardless of kernel type. This group consisted primarily of NAM lines (*n* = 19), including 13 tropical lines, along with two Bulgarian lines from the CNU subpopulation. The separation of this group suggests a distinct genetic background likely attributable to shared ancestral lineage rather than kernel phenotype or panel designation. Collectively, the whole panel (*n* = 168) was partitioned into four major genetic groups. Most clusters were largely consistent with kernel type, whereas one subgroup comprised a subset of the NAM founders that was genetically distinct despite sharing the same kernel type with other accessions.

### 3.3 SNP significance in GWAS generally increased with subpopulation integration

Using the RDA panel as the core population, GWAS was conducted by progressively integrating smaller subpopulations to expand sample size. Across four population combinations analyzed using FarmCPU and BLINK, a total of 33 SNPs exceeding the suggestive threshold (5.73) in either FarmCPU or BLINK were identified for eight kernel‐related traits. Within ±100 kb of these loci, 145 candidate genes were annotated (Figures 3 and 4 and, Figures S5 and S6; Tables S5 and S6).

**FIGURE 3:**
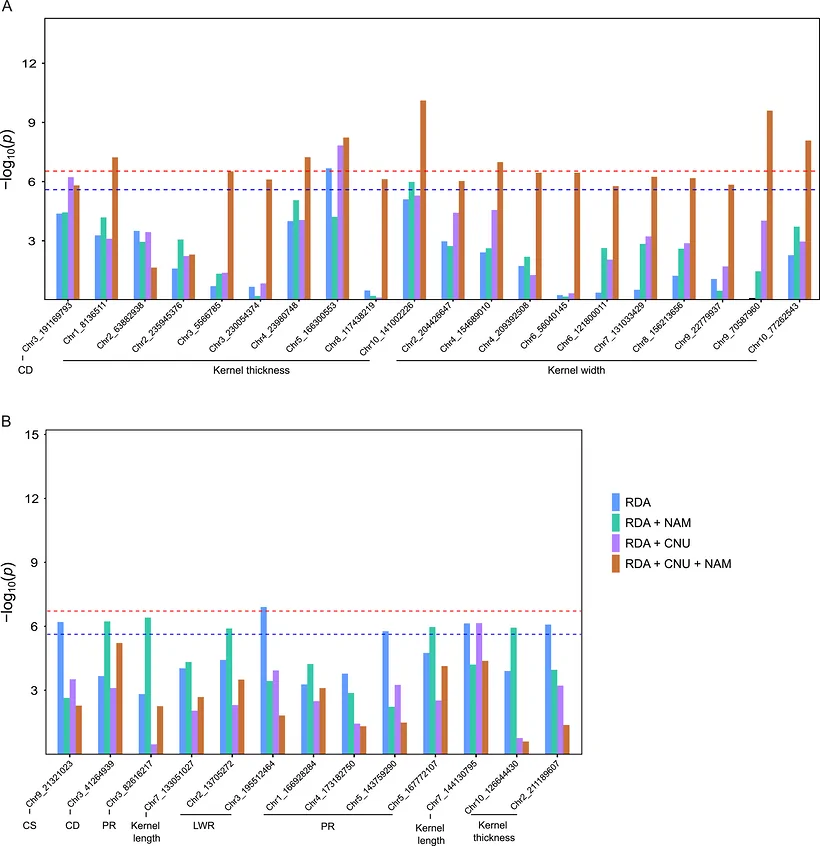
Statistical significance (−log_10_(*p*)) of 33 identified single‐nucleotide polymorphisms (SNPs) across four population combinations using the FarmCPU model. The red and blue dashed lines indicate the suggestive (5.73) and SimpleM significance threshold (6.52), respectively. Text labels below the SNPs represent the traits associated with each SNP. These SNPs were identified as at least one of the two genome‐wide association study (GWAS) models (FarmCPU or BLINK) and four combinations (Rural Development Administration [RDA], *n* = 93; RDA + Nested Association Mapping [NAM], *n* = 119; RDA + Chungnam National University [CNU], *n* = 142; RDA + CNU + NAM, *n* = 168). (A) Statistical significance (−log_10_(*p*)) of 20 SNPs identified in the whole population (*n* = 168) GWAS. (B) Statistical significance (−log_10_(*p*)) of 13 SNPs identified three other population combinations GWAS.

**FIGURE 4:**
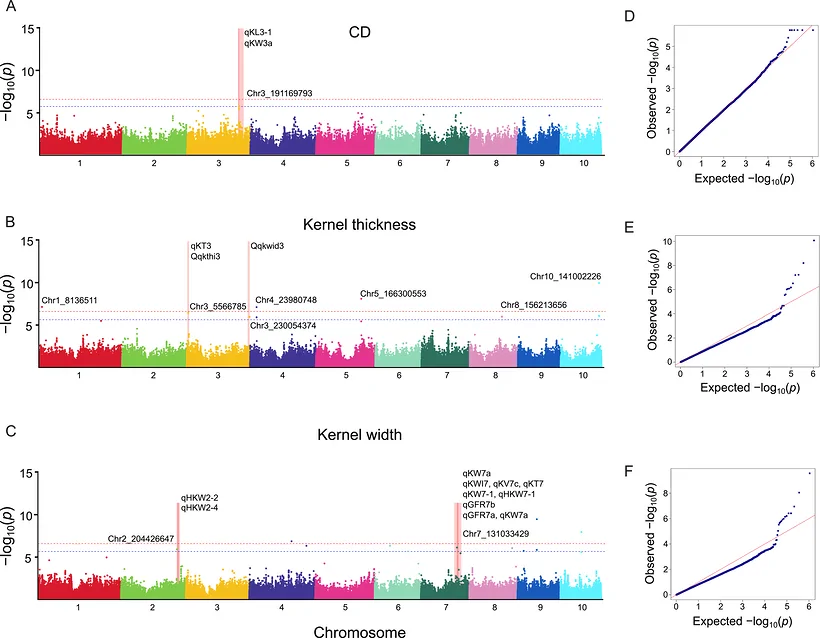
Manhattan plots and *Q–Q* plots (*quantile–quantile* plots) of whole population (*n* = 168) genome‐wide association study (GWAS) on three traits (CD: Centroid deviation, kernel thickness, and kernel width) that quantitative trait locus (QTL) overlapped single‐nucleotide polymorphisms (SNPs) identified by FarmCPU model. (A–C) Manhattan plot of three traits. Intervals colored red in the middle of Manhattan plot represent the range of kernel‐related QTLs on chromosomes. Text labels of nearby colored intervals and SNPs represent its name from previous publications and in this paper, respectively. Among the 20 identified SNPs, only 10 loci that overlapped with or were located close to QTL intervals were labeled with text. For CD, a cluster of six SNPs was identified within the same genomic region, and these SNPs showed identical association strength. Also, these six SNPs were overlapped by QTL interval. The red and blue dashed lines indicate the suggestive (5.73) and SimpleM significance threshold (6.52), respectively. (D–F) *Q–Q* plot of each trait.

We next compared the statistical significance of the 33 SNPs across population combinations. Among the 20 SNPs identified in the whole‐population GWAS, most exhibited increasing −log_10_(*p*) values as population size expanded. In smaller or similar‐sized combinations, these SNPs often did not exceed the suggestive threshold; however, their signals generally and progressively strengthened with increasing sample size, with some showing marked increases in the whole‐population GWAS (Figure 3A and Figure S6A; Table S6).

Three SNPs that were detected from the whole population were identified across multiple combinations despite smaller sample sizes, but each displayed a distinct pattern. For example, both *Chr3_191169793* and *Chr5_166300553* showed strong association signals in the RDA + CNU combination in all three GWAS models, while their signal strengths decreased following the inclusion of NAM. In particular, *Chr5_166300553* showed more pronounced changes in signal strength depending on the inclusion of NAM founders, with a decrease from RDA to RDA + NAM followed by a recovery from RDA + NAM to RDA + CNU, except in the MLM model. Unlike *Chr3_191169793*, *Chr5_166300553* ultimately showed its strongest association signal in the whole population. In contrast, *Chr10_141002226* was consistently identified in combinations including NAM and, similar to *Chr5_166300553*, it showed its strongest association signal in the whole‐population GWAS.

The remaining 13 SNPs, identified primarily in smaller population combinations, generally exhibited strong signals only within the specific combination in which they were identified. In many cases, their significance declined when an additional subpopulation was incorporated (Figure 3B and Figure S6B; Table S6). For example, SNPs identified in RDA or RDA + CNU often showed reduced strength of the signal after adding NAM, despite increased sample size. In the case of *Chr7_144130795*, although identified in two combinations and across three traits, displayed diminished strength of the signal in NAM‐containing combinations. Conversely, SNPs identified in the RDA + NAM combination showed substantially reduced strength of the signal in the RDA + CNU combination, whereas some of these SNPs exhibited a partial recovery of strength of the signal in the whole population. These tendencies were consistently observed in both smaller population and the whole‐population combination (Figure 3B and Figure S6B; Table S6), suggesting that genetic backgrounds and associated phenotypic variations of integrated subpopulations (Figures 1 and 2A,C) can clearly impact overall GWAS outcomes.

Collectively, these results demonstrate that expanding population size through subpopulation integration enhances the identification of novel SNP associations. However, the strength of specific association signals depends not only on sample size but also on the genetic composition of the combined populations, indicating that subpopulation structure influences GWAS outcomes.

### 3.4 SNP statistical significance after controlling genetic background with MLM

The detection of GWAS signals can be influenced by multiple factors, including population size, allele frequency, phenotypic distribution, and genetic background. In particular, when additional subpopulations are incorporated into a GWAS panel, allele frequencies may shift depending on the allelic composition of the added subpopulation, potentially affecting the detection and strength of association signals. To further examine whether the SNPs identified by FarmCPU and BLINK were associated with changes in allele frequency or with background‐dependent allelic effects, we conducted an additional GWAS analysis for each population combination, using a MLM that accounts for population structure and kinship (Hoffman, 2013; Z. Zhang et al., 2010).

Across the 33 putative SNPs identified by FarmCPU and BLINK, overall SNP strength of the signal was reduced in the MLM analysis; however, the patterns of strength of the signal across population combinations were generally consistent with those observed in the FarmCPU and BLINK analyses (Figure S7; Table S6). However, allele frequencies showed relatively limited changes after the addition of subpopulations (Table S7). Using MLM‐derived GWAS statistics, including additive‐effect estimates for each trait (Table S8), we compared the correlations of −log_10_(*p*) values with allele frequency and absolute additive‐effect estimates for each SNP across the population combinations. For the 20 SNPs detected in the whole population, Pearson's correlation coefficients (*r*) between absolute additive‐effect estimates and −log_10_(*p*) values were consistently moderate to high across population combinations, with an average absolute correlation of approximately 0.71. In contrast, correlations between allele frequency and −log_10_(*p*) values were lower and less consistent, ranging from −0.02 to 0.52, with an average absolute correlation of approximately 0.3 (Figure S8A–H). These results suggest that, for these 20 SNPs, variation in SNP association strength across population combinations was more closely associated with changes in estimated additive effects than with changes in allele frequency. In contrast, the 13 SNPs identified in smaller population combinations showed relatively weak correlations of −log_10_(*p*) values with both absolute additive‐effect estimates and allele frequency, with mean absolute correlations of approximately 0.35 and 0.21, respectively (Figure S8I–P). Therefore, the changes in statistical strength of these SNPs across population combinations could not be fully explained by allele frequency or additive‐effect estimates alone.

Collectively, these results indicate that, after accounting for population structure and kinship using the MLM, the overall SNP statistical strength of the 33 SNPs was attenuated, with only four SNPs exceeding the suggestive threshold. Among these four SNPs, only one SNP (*Chr10_141002226*) exceeded the SimpleM‐derived significance threshold (6.52). However, the relative patterns of SNP association strength across population combinations remained broadly consistent. For the 20 putative SNPs, changes in SNP association strength across population combinations were more closely associated with variation in additive‐effect estimates than with allele‐frequency shifts.

### 3.5 SNPs identified across four different combinations overlapped with previously reported maize kernel‐related QTLs

We next examined whether the identified SNPs co‐localized with previously reported maize kernel‐related QTLs (Chen et al., 2016; Jiang at el., 2015; Lan et al., 2018; Lu et al., 2006; Liu et al., 2014; Peng et al., 2011; Z. Zhang et al., 2013; Z. Zhang et al., 2014). A total of 151 published QTL intervals were compiled, and their genomic coordinates were determined in our reference genome using BLAST‐based alignment. In addition to direct overlaps, we also examined whether the identified SNPs were located near previously reported QTL regions, because extending the genomic window around GWAS‐identified loci may facilitate causal gene discovery (Qi et al., 2025). Considering the population structure and genetic background of our GWAS panel (Figure S4A,B), we adopted a more conservative window of ±0.5 Mb around each reported QTL region. As a result, 12 SNPs directly overlapped with previously reported QTL intervals, and one additional SNP was located within 0.5 Mb of QTL regions, resulting in a total of 13 candidate SNPs for further investigation (Table S9).

Among SNPs identified in the whole‐population GWAS, one CD‐associated SNP (*Chr3_191169793*), two kernel thickness‐associated SNPs (*Chr3_5566785* and *Chr3_230054374*), and two kernel width‐associated SNPs (*Chr2_204426647* and *Chr7_131033429*) overlapped with 2, 3, and 7 QTL intervals, respectively. In addition, one kernel thickness‐associated SNP was located near three QTL intervals in the range of 0.28–0.42 Mb (Figure 4A–C; Table S9). Across the different population combinations, seven SNPs overlapped QTL intervals. These SNPs were associated not only with CD, kernel thickness, and kernel width, but also with AS, CS, kernel length, LWR, and PR (Figure 3B; Table S9), indicating that conducting GWAS across various population combinations enables the identification of genetic variants across a broad range of traits.

Notably, several SNPs were physically proximal and shared QTL intervals despite being identified in different population combinations or associated with different traits. For example, *Chr7_131033429* and *Chr7_133051027* overlapped or were close to four QTL intervals and were associated with kernel length and kernel width, respectively—traits that exhibit moderate phenotypic correlation. Similarly, *Chr3_191169793* (CD) and *Chr3_195512464* (LWR) shared the QTL interval qKW3a and corresponded to moderately correlated traits (Figure 1J; Table S9). Moreover, a single SNP, *Chr7_144130795*, consistently identified across three traits (AS, kernel length, and PR) and two population combinations overlapped four previously reported kernel‐related QTLs (Figure 1J and Figure S9).

Collectively, 13 identified SNPs overlapped with or were located near previously reported kernel‐related QTLs. The consistency of shared QTL intervals or identification of common SNPs across multiple traits and population combinations indicates that integrating smaller subpopulations enhances GWAS detection power. These candidate loci likely harbor pleiotropic genes or key regulators influencing diverse aspects of kernel morphology and therefore merit further functional validation.

### 3.6 Single‐SNP genotype effects within LD blocks reveal trait‐specific and pleiotropic associations

Among the 33 SNPs that exceed the suggestive threshold in GWAS, we first prioritized 20 SNPs detected in whole‐population GWAS because they showed strong correlations (*r* ≥ 0.7) between absolute additive‐effect estimates and −log_10_(*p*) values in the MLM, which accounts for population structure and kinship (Hoffman, 2013; Z. Zhang et al., 2010). We then filtered two candidate SNPs among these 20 SNPs that overlapped with or were proximal to previously reported QTLs for the same or related traits and finally exceeded the SimpleM significance threshold (6.52) (Figure 3 and Figures S6–S8; Tables S2, S6, and S9). We then investigated LD patterns around these loci. At the kernel width‐associated locus on chromosome 2, two adjacent LD blocks separated by 185 bp were merged into a single unit because both overlapped genic regions (Figure 5A). The lead SNP showed significant single‐SNP genotype effects on AS, PR, kernel length, and kernel width. Although highly correlated traits were affected, the strongest association was observed for kernel width, suggesting a role in overall kernel development (Figure 5C). For the kernel thickness‐associated locus on chromosome 1, LD blocks (10 kb) fully encompassed *Zm00001eb002960* with lead SNPs located within coding regions (Figure 5B). Unlike the kernel width‐associated locus, single‐SNP genotype effects were largely restricted to kernel thickness (Figure 5D), indicating more trait‐specific genetic control.

**FIGURE 5:**
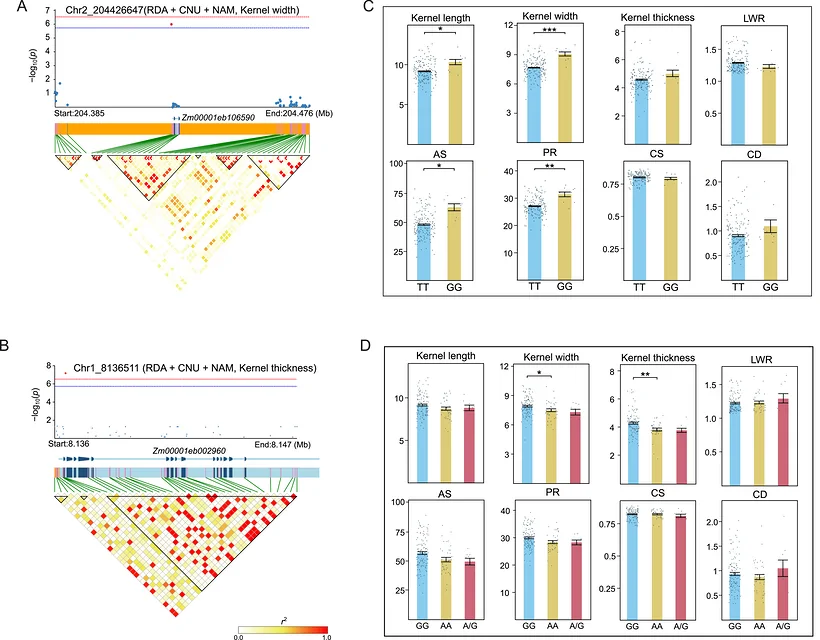
Linkage disequilibrium (LD) block and single‐nucleotide polymorphism (SNP) genotype‐based phenotype comparison of four identified SNPs. (A, B) Association mapping and pairwise LD analysis of genomic regions around four identified SNPs. The red and blue dashed lines indicate the suggestive (5.73) and SimpleM significance threshold (6.52), respectively. Red and blue dots represent the positions of SNPs exceeding the suggestive threshold (5.73) and those located in their genomic proximity, respectively. Rectangular bars, marked with multiple colors and lines, highlight specific points corresponding to SNPs (pink), intergenic regions (orange) introns (light bule), and exons (dark blue) within the genomic regions. Black triangles in the lower panel highlight SNPs that exhibit high LD with the lead variant. (C, D) Single‐SNP genotypes of each variant and their phenotypic distribution on eight kernel‐related traits. Scales for *y*‐axis are same as Figure 1A–H. Bars represent the mean, and error bars indicate the standard error (SE). Statistical significance is indicated by asterisks: *p < 0.05, **p < 0.01, and ***p < 0.001.

In the case of *Chr7_144130795*, although this SNP was not included among the 20 SNPs detected in the whole‐population GWAS (*n* = 168) and did not exceed the SimpleM threshold (6.52), it was consistently identified across multiple population combinations and kernel size‐related traits (kernel length, AS, and PR). Moreover, its association strength exceeded the suggestive threshold (5.73) in all three GWAS models and overlapped with three previously reported QTLs (Figure 3B and Figures S6B, S7B, and S9; Table S9). Around this position, a 7 kb LD block containing *Zm00001eb319930* was identified (Figure S10A). The lead SNP resided in the fifth intron. SNP genotype‐based phenotype comparisons showed significant differences for AS and PR, and with the strongest effect observed for kernel width (Figure S10C). Same with *Chr7_144130795*, the CD‐associated locus *Chr3_191169793* (highest −log_10_(*p*) in the RDA + CNU combination) did not exceed its association strength at the SimpleM threshold (6.52) but maintained its strength in all three GWAS models and two population combinations (RDA + CNU and whole population). Around this position, two LD blocks were identified: a 74 kb block (37 SNPs) and a 36 kb block (25 SNPs). These blocks encompassed *Zm00001eb151510* and *Zm00001eb151530*. The highest SNPs in their association strength were intronic within these genes, and one SNP was located between adjacent LD blocks. We therefore defined an expanded candidate interval spanning the LD blocks and intervening region (Figures 3A and 4A, and Figure S10B). SNP genotype‐based phenotype comparison analysis revealed significant differences not only for CD but also for AS, CS, kernel length, LWR, and PR (Figure S10D). Given the moderate correlations between CD and these traits (Figure 1J), this locus likely exerts pleiotropic effects on kernel morphology.

Collectively, these results indicate that the LD blocks containing the candidate SNPs represent functionally relevant genomic units. Among the candidates, *Zm00001eb106590*, *Zm00001eb151510*, and *Zm00001eb319930* are strong kernel‐related genes, whereas *Zm00001eb002960* appears to regulate kernel thickness in a more trait‐specific manner.

## 4 DISCUSSION

### 4.1 Integration of subpopulations influences GWAS signal detection

This study evaluated whether integrating small maize subpopulations improves GWAS performance for kernel‐related traits. Expanding population size generally improved the detectability of association signals and enabled detection of loci that were not identifiable in smaller panels. However, because the final sample size of 168 remains relatively limited for GWAS, these results should be interpreted cautiously and validated in larger independent populations. Most SNPs identified in the whole population showed progressively higher −log_10_(*p*) values as sample size increased, supporting the principle that larger populations enhance statistical power and mapping resolution (Figure 6A; Atwell et al., 2010; Lell et al., 2024).

**FIGURE 6:**
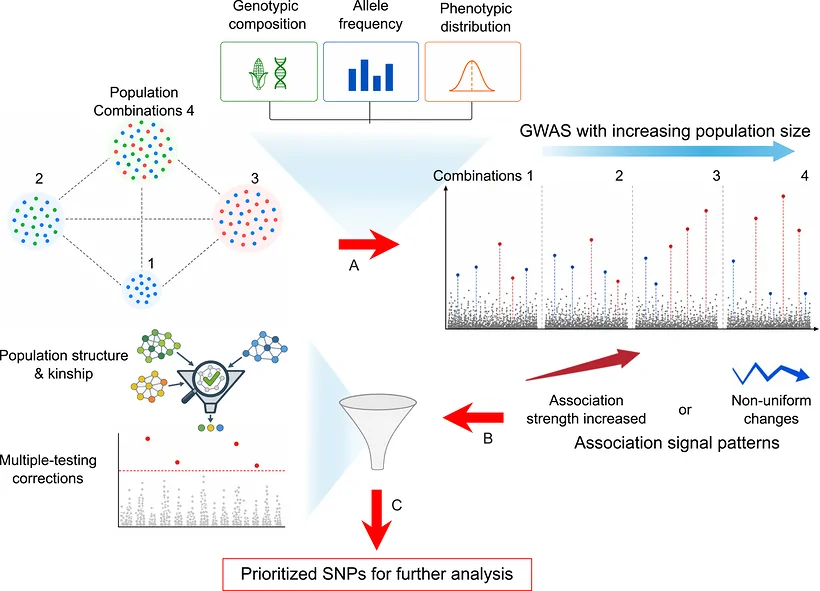
Conceptual illustration of the strategic integration of small populations for genome‐wide association study (GWAS). (A) Small populations are progressively combined while considering genotypic composition, allele frequency, and phenotypic distribution, followed by GWAS with increasing population size. Increasing population size may strengthen association signals, although changes in association strength may not be uniform across loci. (B) Association signals are evaluated together with appropriate control of population structure and kinship and multiple‐testing correction to reduce potential confounding and false‐positive associations. (C) single‐nucleotide polymorphisms (SNPs) supported through these evaluations are prioritized for further analysis. This approach emphasizes that effective population integration should consider both population compatibility and association‐signal stability rather than sample‐size expansion alone

However, increased sample size did not uniformly strengthen all associations. While 20 SNPs were consistently detected and showed enhanced association strength in the whole‐population GWAS, 13 SNPs identified in smaller population combinations lost association strength after additional subpopulations were incorporated (Figure 3 and 6B). This is consistent with previous study suggesting that the phenotypic expression of an allele is not a simple direct result but is significantly shaped by its interaction with the genetic background, which can lead to the masking or attenuation of genetic signals in diverse populations (Chandler et al., 2013). This indicates that GWAS power depends not only on population size but also on phenotypic distribution and genetic background composition (Figure 6B). Admixture analysis revealed clear genetic differentiation between the NAM founders, which largely consist of tropical lines, and the RDA and CNU populations (Figure 2C; Hufford et al., 2021; J. Yu et al., 2008). Although LD decay patterns were similar among the three panels (Figure S2A), phenotypic distributions, particularly for size‐related traits (kernel length, kernel width, kernel thickness, AS, and PR), differed among subpopulations (Figure 1A–H). However, the phenotypic datasets had insufficient genotype overlap across seasons to reliably adjust for systematic environmental variation with a best linear unbiased prediction‐based mixed model. Therefore, unaccounted environmental effects may have contributed to the observed phenotypic variation and should be considered when interpreting the GWAS results. In addition, such background‐dependent variation likely influenced allele effects, leading to context‐specific detection patterns. Consistent with this, in genetic association analysis, heterogeneity in phenotypic variance caused by genetic background may attenuate or even obscure true associations, leading to divergent analysis results across populations and individuals (Deng et al., 2017; Liao et al., 2014; Sanchez‐Bermejo et al., 2015; Wei et al., 2024; Yadav et al., 2019). These results suggest that the benefit of increasing population size through subpopulation integration depends on the characteristics of the populations being combined. Therefore, genotypic composition, allele‐frequency differences, and phenotypic distribution should be considered when combining subpopulations (Figure 6A).

Nevertheless, the effect of incorporating genetically divergent populations was not unidirectional. In several cases, the inclusion of NAM founders increased the strength of the signal, likely because larger sample sizes and broader phenotypic variation can improve the power to detect genetic associations (Figure 3B and Figure S6B). Furthermore, by combining the RDA, CNU, and NAM founders, we evaluated how differences in genetic background and phenotypic distribution influence GWAS signal detection. This approach enabled us to determine whether association signals were strengthened or attenuated following the incorporation of genetically distinct subpopulations. However, this should not be interpreted as suggesting that population composition should be selectively manipulated to maximize GWAS signal strength. Rather, subpopulation integration should be guided by careful consideration of genome‐wide genetic structure, genetic distance, LD patterns, phenotypic distribution, allele‐frequency distribution, and trait‐relevant diversity. Overall, our results suggest that strategic GWAS panel design requires balancing increased sample size and phenotypic diversity against the potential confounding effects of genetic‐background heterogeneity.

### 4.2 Potential model–population interaction signals associated with subpopulation integration

In this study, a total of 33 SNPs exceeded the suggestive threshold across the four population combinations in either the FarmCPU or BLINK model (Figure 3 and Figure S6; Table S6). However, the association strength of most of these SNPs was reduced in the MLM analysis, and many no longer exceeded the suggestive threshold (Figure S7; Table S6). Nevertheless, most SNPs retained their highest MLM‐based signal strength in the population combination in which they were originally identified. In contrast, some SNPs showed a marked reduction in association strength after applying the MLM.

For example, among the 20 SNPs identified in the whole‐population GWAS, six SNPs—*Chr3_191169793*, *Chr5_166300553*, *Chr10_141002226*, *Chr2_204426647*, *Chr9_70587960*, and *Chr10_77262543*—exceeded the suggestive threshold in more than one GWAS model. In contrast, the remaining SNPs exceeded the threshold in only one of the two models, FarmCPU or BLINK. Among these model‐specific SNPs, six SNPs—*Chr3_5566785*, *Chr3_230054374*, *Chr8_117438219*, *Chr4_209392508*, *Chr6_56040145*, and *Chr9_22779937*—showed substantially reduced association strength in the MLM analysis, whereas eight SNPs—*Chr1_8136511*, *Chr2_63882938*, *Chr2_235945376*, *Chr4_23980748*, *Chr4_154689010*, *Chr6_121800011*, *Chr7_131033429*, and *Chr8_156213656*—retained relatively higher MLM‐based association strength (Figure S7A; Table S6). Among the 13 SNPs identified in the other three population combinations, three SNPs—*Chr1_166928284*, *Chr4_173182750*, and *Chr7_133051027*—were detected in only one GWAS model and showed reduced association strength after applying the MLM panel they detected from the other GWAS model (Figure S7B; Table S6).

Previous studies have suggested that SNP associations can differ depending on ancestry or population background, which may be interpreted as gene–ancestry interactions, ancestry‐specific associations, or genotype‐by‐population interaction effects (Davies et al., 2012; C. Yu et al., 2020). In admixed or structured populations, population substructure, ancestry composition, and local LD differences may influence association signals and complicate their interpretation. Therefore, subpopulation‐specific peaks should be interpreted cautiously and may not always represent universal main‐effect loci but may also reflect background‐dependent associations (Davies et al., 2012; C. Yu et al., 2020). In this context, the MLM provides a complementary framework for evaluating whether signals remain detectable after accounting for population structure and kinship (Hoffman, 2013; Z. Zhang et al., 2010). In addition to model‐based evaluation, the detected associations were further assessed using the SimpleM‐derived significance threshold, which accounts for LD by estimating the effective number of independent markers and therefore provides an additional criterion for evaluating statistical support under multiple testing (R. C. Johnson et al., 2010; Tibbs‐Cortes et al., 2021). Accordingly, signals supported by the SimpleM‐derived threshold were considered to have stronger statistical support, whereas associations exceeding only the suggestive threshold were interpreted more cautiously.

These observations suggest that SNPs detected by only one GWAS model in this analysis and attenuated in the MLM analysis may represent model‐sensitive signals, although further validation is required to determine whether they are influenced by population‐specific genetic backgrounds. Moreover, SNPs showing lower association strength in the MLM than in FarmCPU or BLINK tended to show panel‐specific increases in association strength, particularly in panels including NAM. Because NAM contained genetically distinct subgroups based on population structure analysis, changes in population structure following subpopulation integration may have contributed to differences in signal detection among GWAS models (Figure 2B,C and Figures S4E and S7). Thus, SNPs showing consistent or relatively robust association signals across complementary GWAS models, particularly those retaining support after accounting for population structure and kinship and showing stronger statistical support according to the SimpleM‐derived threshold, can be prioritized for subsequent biological interpretation and further validation (Figure 6C).

### 4.3 Single‐SNP genotype and maize kernel‐related QTLs are associated with multiple traits

Kernel‐related traits such as length, width, and thickness are genetically interconnected, and previous QTL studies have shown that grain traits often influence one another (J. Liu et al., 2017; L. Ma et al., 2016; X. Zhang et al., 2012). In this study, we performed GWAS on eight kernel‐related traits and compared our prioritized SNPs with previously reported maize kernel QTLs. Many overlapping or nearby QTLs corresponded to traits different from those identified in our GWAS, highlighting the multi‐trait nature of these genomic regions.

For example, the kernel width‐associated SNP *Chr2_204426647* overlapped with QTLs for 100‐kernel weight, and its single‐SNP genotypes showed significant differences in four traits (Figures 1J, 4, and 5; Table S6). Similarly, *Chr7_144130795* was repeatedly identified across multiple traits and population combinations, and its genotypes displayed phenotypic differences largely consistent with the associated traits, although some trait‐specific discrepancies were observed (Figures S9 and S10A,C). Also, the region overlapping *Chr3_191169793* contains QTLs associated with kernel length and width, and its genotypes exhibited significant phenotypic differences across six traits (Figure S10B,D).

In contrast, *Chr1_8136511* showed strong trait specificity. The overlapping QTLs and SNP genotypes consistently corresponded to kernel thickness, and it exhibited low phenotypic correlation with other traits. This suggests that this locus may function as more direct and independent regulators of thickness, as opposed to the other locus. Moreover, this tendency is consistent with the phenotypic correlations among the eight traits, where kernel thickness shows low correlation with the other seven (Figures 1J, 4B, and 5D).

Overall, most GWAS‐identified SNPs were located in genomic regions harboring multi‐trait kernel QTLs, reflecting the interconnected genetic architecture of kernel morphology. However, integrating GWAS results with previously reported QTL information enabled the identification of trait‐specific loci, particularly for kernel thickness.

### 4.4 Maize kernel‐related candidate genes identified from different population combinations

From different population combinations, several biologically plausible candidate genes were identified within LD blocks surrounding prioritized SNPs. For *Chr2_204426647*, we identified *Zm00001eb106590* annotated as pentatricopeptide repeat (PPR) proteins. PPR genes are well‐known regulators of organellar RNA processing, influencing photosynthesis, respiration, and overall plant growth (Barkan & Small, 2014; Wang & Tan, 2025). In cereals, PPR genes have been repeatedly associated with grain development, yield components, and grain quality. Studies in wheat and rice have linked PPRs to grain number, kernel weight, seed size, endosperm development, and starch accumulation (Hao et al., 2019; Hou et al., 2025; Wu et al., 2019; H. Yang et al., 2023). Similarly, in maize, mutations in PPR genes frequently result in defective endosperm development, reduced grain filling, and decreased kernel weight (Huang et al., 2020; S. Ma et al., 2022; Zang et al., 2024; Zhu et al., 2019). These previous findings strongly support the candidacy of *Zm00001eb106590* as regulators of kernel‐related traits.

*Chr7_144130795* and *Chr3_191169793* were consistently identified across several GWAS combinations, although their association strength weakened as population size increased. Despite this, LD‐preserved regions surrounding these SNPs contained candidate genes overlapping previously reported kernel‐related QTLs. At *Chr3_191169793*, we identified *Zm00001eb151510* and *Zm00001eb151530*, annotated as insulin‐degrading enzyme‐like 1, peroxisomal, and vacuole membrane protein KMS1, respectively. Insulin‐degrading enzymes are conserved oligopeptidases involved in protein turnover and metabolic regulation, with localization reported in cytosol, mitochondria, and peroxisomes (Corraliza‐Gómez et al., 2022). In other plant systems, this enzyme has been implicated in metabolic regulation during fruit development, and in maize it has been prioritized as a stress‐responsive core gene (Pradhan et al., 2025). Vacuole membrane protein KMS1 is an endoplasmic reticulum (ER)‐associated protein that plays a critical role in maintaining ER structural integrity and mediating the early secretory pathway from the ER to the Golgi apparatus in plant (P. Wang et al., 2011). However, the functional role of KMS1 in kernel development remains poorly understood and has not been extensively studied. Given their potential roles in stress response and metabolic regulation, *Zm00001eb151510* and *Zm00001eb151530* may exert pleiotropic effects on kernel development, consistent with the significant genotype‐dependent differences observed across multiple traits.

For *Chr1_8136511*, the candidate gene *Zm00001eb002960* was annotated as aconitase. In maize, aconitase serves as a key catalytic enzyme within the tricarboxylic acid cycle, facilitating the isomerization of citrate to isocitrate to support cellular energy production and carbon skeleton supply (Y. Liu et al., 2019; N. Zhang et al., 2010). This enzyme exhibits high expression in male reproductive organs, such as anthers and tassels, to meet the substantial energy demands required for microspore development and fertility (Wen et al., 2015). Furthermore, it is vital for kernel and root development, where its activity in the mitochondria is intrinsically linked to iron (Fe) homeostasis and Fe–sulfur (S) cluster stability, thereby influencing overall plant growth and stress resilience (Zang et al., 2020).

For *Chr7_144130795*, *Zm00001eb319930* was identified, encoding a peptidyl‐prolyl cis–trans isomerase (PPIase). Although PPIases have not been extensively studied in relation to grain traits, they function in protein folding and ER quality control. In maize endosperm, PPIase activity has been detected among ER‐associated chaperones involved in storage protein assembly. In wheat, PPIase activity correlates with storage protein accumulation during grain development (Gutierrez & Torres, 2025; Dutta et al., 2011). The repeated detection of *Chr7_144130795* from GWAS across multiple traits (AS, PR, and kernel length), together with significant genotype‐dependent phenotypic differences, suggests that *Zm00001eb319930* may contribute to variation in kernel morphology.

Overall, integrating GWAS signals from different population combinations with LD and QTL information enabled the identification of biologically meaningful candidate genes. Several loci point to conserved pathways related to organellar function, protein turnover, and protein folding, underscoring the complex molecular regulation underlying maize kernel development.

## 5 CONCLUSION

Our findings suggest that the integration of small maize populations has the potential to improve GWAS detection power while retaining meaningful association signals. Importantly, the effects of population integration were not uniform across loci, and the effectiveness of population integration depends on the genetic and phenotypic compatibility of the populations being combined. In particular, genotypic composition, allele‐frequency differences, and phenotypic distribution should be carefully evaluated. Association strength can be assessed using appropriate population structure and kinship, together with multiple‐testing correction (Figure 6). This methodological approach provides practical guidance for the effective use of multiple small populations and may facilitate the construction of more informative GWAS panels when the development of a single large mapping population is impractical.

## AUTHOR CONTRIBUTIONS

**Myeong‐Geon Seok**: Conceptualization; data curation; investigation; resources; visualization; writing—original draft. **Seongmin Hong**: Conceptualization; data curation; visualization; writing—original draft. **Jiyun Go**: Investigation; resources. **Jin Seong Park**: Investigation; resources. **Hobin Lee**: Methodology; resources. **Sanghak Lee**: Investigation. **Gibum Yi**: Conceptualization; funding acquisition; project administration; supervision; validation; writing—original draft; writing—review and editing.

## CONFLICT OF INTEREST STATEMENT

The authors declare no conflicts of interest.

## Supporting information




**Supplemental Figure S1**. Schematic overview and validation of *SmartGrain 1*‐based maize kernel trait measurements. **A**, Representative maize kernel image analyzed using *SmartGrain 1*. The kernel boundary (perimeter) was automatically detected and marked with a red outline. The length and width axes, their intersection point (IS), and the center of gravity (CG) are indicated. CD is the distance between IS and CG. Scale bar = 0.5 cm. **B**, Formula used to calculate kernel circularity (CS). Circularity was calculated from projected kernel area size (AS) and perimeter length (PR) as CS = 4π × AS / PR^2^. **C**, Scatter plot comparing manually measured kernel length and *SmartGrain 1*‐derived kernel length. **D**, Scatter plot comparing manually measured kernel width and *SmartGrain 1*‐derived kernel width. In C and D, the x‐axis represents manual measurements, and the y‐axis represents *SmartGrain 1* measurements. Pearson correlation analysis was performed using paired manual and *SmartGrain 1* measurements, and (*r*) was calculated from the Pearson correlation coefficient. The red dashed line indicates the line of perfect agreement, y = x, and the blue line indicates the linear regression line.
**Supplemental Figure S2**. Scatterplot matrix showing pairwise relationships among eight kernel‐related traits. The upper panels indicate Pearson's correlation coefficients with significance levels, the lower panels show pairwise scatterplots, and the blue lines represent fitted linear regression lines showing the linear trend between each pair of traits. The diagonal panels show the distribution of each trait.
**Supplemental Figure S3**. Phenotypic distribution of four population combinations and kernel types for eight kernel‐related traits. The RDA, CNU, and NAM subpopulations consisted of 93, 49, and 26 accessions, respectively. Seven kernel traits were measured using the grain analysis program, *SmartGrain 1* and thickness was measured by digital Vernier calipers. Statistically significant differences (p < 0.05) among combinations are denoted by distinct letters above the boxes, as determined by Duncan's Multiple Range Test (DMRT). **A‐H**, While significant phenotypic differences were observed among subpopulations, no significant differences were found among their combinations. **I‐P**, Phenotypic distributions of kernel types on eight kernel‐related traits. The number of accessions in each kernel type was: 71 flint, 29 dent, 49 waxy, 18 sweet, 1 popcorn; the popcorn was excluded from the figure due to its insufficient sample size. Waxy kernels often exhibit a globular form, distinct from the elliptical shapes typical of other types. This morphological difference is reflected in marked variations in shape‐related traits (CS, LWR, and CD). The tendency toward sphericity in waxy maize appears to result from the differential regulation of kernel length relative to other size‐related traits.
**Supplemental Figure S4**. Genetic population structure, cross‐validation, and LD patterns according to subpopulation, kernel type, and population combination. **A**, LD decay of three subpopulations. The LD decay patterns across the three populations (CNU, NAM, and RDA) are highly comparable, showing minimal variation in their rates of decay. **B**, LD decay patterns of four kernel types. While the LD decay patterns were relatively uniform among subpopulations, they exhibited significant divergence when separated by kernel types. **C**, Population structure of 168 accessions at K = 3 integrated with their phylogenetic relationship. The ADMIXTURE bar plot is aligned with the topology of the maximum‐likelihood phylogenetic tree. Each vertical bar represents an accession, and colored segments indicate the proportional contribution of the four ancestral populations. The colored horizontal bar and dots above and below the plot indicate the subpopulation assignment and kernel type of each line, respectively. **D‐F**, Cross‐validation (CV) error plots from ADMIXTURE analysis for population combinations of D (n = 93), E (n = 119), and F (n = 142). K represents the number of assumed ancestral populations (K = 1–10), and 10 independent replicates were performed for each K to estimate the CV error.
**Supplemental Figure S5**. Manhattan plots and Q‐Q plots for five traits in the whole population (n = 168) GWAS using FarmCPU model. **A‐E**, Manhattan plots of five traits. The red and blue dashed lines indicate the suggestive (5.73) and simpleM significance threshold (6.52) respectively. No SNPs exceeded the suggestive threshold for these five traits. **F‐J**, Q‐Q plot of each trait.
**Supplemental Figure S6**. Statistical significance (−log_10_(*p*)) of the 33 identified SNPs across four population combinations in the BLINK model. The red and blue dashed lines indicate the suggestive (5.73) and simpleM significance threshold (6.52) respectively. Text labels below the SNPs represent the traits associated with each SNP. These SNPs were identified in at least one of the two GWAS models (FarmCPU or BLINK) and in at least one of the four population combinations (RDA, n = 93; RDA + NAM, n = 119; RDA + CNU, n = 142; RDA + CNU + NAM, n = 168). **A**, Statistical significance (−log_10_(*p*)) of 20 SNPs identified in the whole population (n = 168) GWAS. 11 SNPs did not exceed the suggestive threshold in the BLINK model. **B**, Statistical significance (−log_10_(*p*)) of 13 SNPs identified in GWAS of the other three population combinations. Only three SNP did not exceed the suggestive threshold in BLINK model.
**Supplemental Figure S7**. Statistical significance (−log_10_(*p*)) of the 33 identified SNPs across four population combinations in the MLM. The red and blue dashed lines indicate the suggestive (5.73) and simpleM significance threshold (6.52) respectively. Text labels below the SNPs represent the traits associated with each SNP. These SNPs were identified in at least one of the two GWAS models (FarmCPU or BLINK) and in at least one of the four population combinations (RDA, n = 93; RDA + NAM, n = 119; RDA + CNU, n = 142; RDA + CNU + NAM, n = 168). **A**, Statistical significance (−log_10_(*p*)) of 20 SNPs identified in the whole‐population GWAS (n = 168). 11 SNPs did not exceed the suggestive threshold in the BLINK model. **B**, Statistical significance (−log_10_(*p*)) of 13 SNPs identified in the GWAS of the other three population combinations.
**Supplemental Figure S8**. Correlation between absolute additive effects and MLM‐based SNP significance across population combinations. Scatter plots show the relationship between the absolute additive effect values and MLM‐based statistical significance values (−log_10_(*p*)) for the same SNPs within each population combination: RDA, RDA + NAM, RDA + CNU, and RDA + CNU + NAM. Each point represents one SNP. Additive effect values were converted to absolute values before analysis to evaluate the relationship between effect size magnitude and association strength, regardless of effect direction. Pearson's correlation coefficient (*r*) and corresponding *p*‐value were calculated separately for each population combination. The blue solid line represents the fitted linear regression trend line, and the gray shaded area indicates the 95% confidence interval around the regression line. **A‐H**, Scatter plots showing the relationship between the statistical significance (−log_10_(*p*)), additive effect values, and allele frequency of 20 SNPs identified in the whole‐population GWAS (n = 168). **I‐P**, Scatter plots showing the relationships between association strength (−log_10_(*p*)), additive effect values, and allele frequency for 13 SNPs identified in the GWAS of the other three population combinations.
**Supplemental Figure S9**. Manhattan plots and Q‐Q plots of GWAS analyses for the RDA (n = 93) and RDA + CNU combinations (n = 142) for three traits. **A‐F**, Manhattan plots of the three traits. Intervals colored red in the middle of the Manhattan plot represent the ranges of kernel‐related QTLs on chromosomes. Text labels of nearby colored intervals and SNPs represent their names reported in previous publications and identified in this study, respectively. The red and blue dashed lines indicate the suggestive (5.73) and simpleM significance threshold (6.52) respectively. The SNP *Chr7_144130795* was not identified only when NAM founders were included. **G‐L**, Q‐Q plot of each trait and combination.
**Supplemental Figure S10**. LD block and SNP genotype‐based phenotype comparison of *Chr7_144130795*. **A**, Association mapping and pairwise LD analysis of genomic regions around *Chr7_144130795*. The red and blue dashed lines indicate the suggestive (5.73) and simpleM significance threshold (6.52) respectively. Red and blue dots represent the positions of SNPs exceeding the suggestive threshold and SNPs located nearby, respectively. Rectangular bars, marked with multiple colors and lines, highlight specific points corresponding to SNPs (pink), intergenic regions (orange), introns (light blue), and exons (dark blue) within the genomic regions. Black triangles in the lower panel highlight SNPs that exhibit high LD with the lead variant. The candidate gene, *Zm00001eb319930*, encodes a peptidyl‐prolyl cis–trans isomerase (PPIase) (Table S5). **B**, Genotypes of each variant and their phenotypic distributions on eight kernel‐related traits. Bars represent the mean, and error bars indicate the standard error (SE). Statistical significance is indicated by asterisks: *, p < 0.05; **, p < 0.01; and ***, p < 0.001. While *Chr7_144130795* was associated with AS, PR, and kernel length, single‐SNP genotype analysis revealed significant differences in kernel width, which showed clear differences despite the lack of statistical significance for this SNP in GWAS results.

**Supplemental Table S1**. Biosample ID list of 168 maize accessions used in the previous and the present studies.
**Supplemental Table S2**. Thresholds determined by different correction methods across four population combinations and traits.
**Supplemental Table S3**. Descriptive statistics of the 168 maize inbred lines in each subpopulation.
**Supplemental Table S4**. Descriptive statistics of the 168 maize inbred lines across kernel types.
**Supplemental Table S5**. Candidate genes located around the 33 SNPs identified across four population combinations.
**Supplemental Table S6**. Statistical significance (−log_10_(*p*)) of the 33 SNPs identified by GWAS across four population combinations.
**Supplemental Table S7**. Allele frequencies of the 33 SNPs identified by GWAS across four population combinations.
**Supplemental Table S8**. Additive effects of the 33 identified SNPs across four population combinations in the MLM.
**Supplemental Table S9**. Previously reported maize kernel‐related QTLs and overlapping or nearby SNPs identified in this study.

## Data Availability

All raw resequencing data generated in this study have been deposited in the National Center for Biotechnology Information (NCBI) Sequence Read Archive (https://www.ncbi.nlm.nih.gov/sra) under the BioProject accession number PRJNA1222583.
