# Supplementary material for: Feasibility and pitfalls of combining small subpopulations for genome‐wide association analysis of maize (Zea mays L.) kernel traits
Source: Plant Genome. 2026 Oct 8;19(4):e70319. doi: 10.1002/tpg2.70319 (PMC13650183; doi:10.1002/tpg2.70319)
Supplement: Supplementary file 1 [file TPG2-19-e70319-s002.docx]

**Feasibility and pitfalls of combining small subpopulations for genome-wide association analysis of maize (*Zea mays* L.) kernel traits**

Myeong-Geon Seok^+^, Seongmin Hong^+^, Jiyun Go, Jin Seong Park, Hobin Lee, Sanghak Lee, Gibum Yi*

Department of Bio-Environmental Chemistry, College of Agriculture and Life Sciences, Chungnam National University, Daejeon, South Korea

^+^These authors contributed equally to this work.

^*^For correspondence: gibumyi@cnu.ac.kr; Tel +82-42-821-6738; Fax +82-42-821-6731

**SUPPORTING INFORMATION**

**Supplemental Figure S1.** Schematic overview and validation of *SmartGrain 1*-based maize kernel trait measurements. **A**, Representative maize kernel image analyzed using *SmartGrain 1*. The kernel boundary (perimeter) was automatically detected and marked with a red outline. The length and width axes, their intersection point (IS), and the center of gravity (CG) are indicated. Centroid deviation (CD) is the distance between IS and CG. Scale bar = 0.5 cm. **B**, Formula used to calculate kernel circularity (CS). Circularity was calculated from projected kernel area size (AS) and perimeter length (PR) as CS = 4π × AS / PR². **C**, Scatter plot comparing manually measured kernel length and *SmartGrain 1*-derived kernel length. **D**, Scatter plot comparing manually measured kernel width and *SmartGrain 1*-derived kernel width. In C and D, the x-axis represents manual measurements, and the y-axis represents *SmartGrain 1* measurements. Pearson correlation analysis was performed using paired manual and *SmartGrain 1* measurements, and (*r*) was calculated from the Pearson correlation coefficient. The red dashed line indicates the line of perfect agreement, y = x, and the blue line indicates the linear regression line.
**Supplemental Figure S2.** Scatterplot matrix showing pairwise relationships among eight kernel-related traits. The upper panels indicate Pearson’s correlation coefficients with significance levels, the lower panels show pairwise scatterplots, and the blue lines represent fitted linear regression lines showing the linear trend between each pair of traits. The diagonal panels show the distribution of each trait.
**Supplemental Figure** **S3.** Phenotypic distribution of four population combinations and kernel types for eight kernel-related traits. The RDA, CNU, and NAM subpopulations consisted of 93, 49, and 26 accessions, respectively. Seven kernel traits were measured using the grain analysis program, *SmartGrain 1* and thickness was measured by digital Vernier calipers. Statistically significant differences (p < 0.05) among combinations are denoted by distinct letters above the boxes, as determined by Duncan’s Multiple Range Test (DMRT). **A-H**, While significant phenotypic differences were observed among subpopulations, no significant differences were found among their combinations. **I-P**, Phenotypic distributions of kernel types on eight kernel-related traits. The number of accessions in each kernel type was: 71 flint, 29 dent, 49 waxy, 18 sweet, 1 popcorn; the popcorn was excluded from the figure due to its insufficient sample size. Waxy kernels often exhibit a globular form, distinct from the elliptical shapes typical of other types. This morphological difference is reflected in marked variations in shape-related traits (CS, LWR, and CD). The tendency toward sphericity in waxy maize appears to result from the differential regulation of kernel length relative to other size-related traits.
**Supplemental Figure** **S4.** Genetic population structure, cross-validation, and LD patterns according to subpopulation, kernel type, and population combination. **A**, LD decay of three subpopulations. The LD decay patterns across the three populations (CNU, NAM, and RDA) are highly comparable, showing minimal variation in their rates of decay. **B**, LD decay patterns of four kernel types. While the LD decay patterns were relatively uniform among subpopulations, they exhibited significant divergence when separated by kernel types. **C**, Population structure of 168 accessions at K = 3 integrated with their phylogenetic relationship. The ADMIXTURE bar plot is aligned with the topology of the maximum-likelihood phylogenetic tree. Each vertical bar represents an accession, and colored segments indicate the proportional contribution of the four ancestral populations. The colored horizontal bar and dots above and below the plot indicate the subpopulation assignment and kernel type of each line, respectively. **D-F**, Cross-validation (CV) error plots from ADMIXTURE analysis for population combinations of D (n = 93), E (n = 119), and F (n = 142). K represents the number of assumed ancestral populations (K = 1–10), and 10 independent replicates were performed for each K to estimate the CV error.
**Supplemental Figure S5.** Manhattan plots and Q-Q plots for five traits in the whole population (n=168) GWAS using FarmCPU model. **A-E**, Manhattan plots of five traits. The red and blue dashed lines indicate the suggestive (5.73) and simpleM significance threshold (6.52) respectively. No SNPs exceeded the suggestive threshold for these five traits. **F-J**, Q-Q plot of each trait.
**Supplemental Figure S6.** Statistical significance (−log_10_(*p*)) of the 33 identified SNPs across four population combinations in the BLINK model. The red and blue dashed lines indicate the suggestive (5.73) and simpleM significance threshold (6.52) respectively. Text labels below the SNPs represent the traits associated with each SNP. These SNPs were identified in at least one of the two GWAS models (FarmCPU or BLINK) and in at least one of the four population combinations (RDA, n = 93; RDA + NAM, n = 119; RDA + CNU, n = 142; RDA + CNU + NAM, n = 168). **A**, Statistical significance (−log_10_(*p*)) of 20 SNPs identified in the whole population (n = 168) GWAS. 11 SNPs did not exceed the suggestive threshold in the BLINK model. **B**, Statistical significance (−log_10_(*p*)) of 13 SNPs identified in GWAS of the other three population combinations. Only three SNP did not exceed the suggestive threshold in BLINK model.

**Supplemental Figure S7.** Statistical significance (−log_10_(*p*)) of the 33 identified SNPs across four population combinations in the MLM. The red and blue dashed lines indicate the suggestive (5.73) and simpleM significance threshold (6.52) respectively. Text labels below the SNPs represent the traits associated with each SNP. These SNPs were identified in at least one of the two GWAS models (FarmCPU or BLINK) and in at least one of the four population combinations (RDA, n = 93; RDA + NAM, n = 119; RDA + CNU, n = 142; RDA + CNU + NAM, n = 168). **A**, Statistical significance (−log_10_(*p*)) of 20 SNPs identified in the whole-population GWAS (n = 168). 11 SNPs did not exceed the suggestive threshold in the BLINK model. **B**, Statistical significance (−log_10_(*p*)) of 13 SNPs identified in the GWAS of the other three population combinations.

**Supplemental Figure S8.** Correlation between absolute additive effects and MLM-based SNP significance across population combinations. Scatter plots show the relationship between the absolute additive effect values and MLM-based statistical significance values (−log_10_(*p*)) for the same SNPs within each population combination: RDA, RDA + NAM, RDA + CNU, and RDA + CNU + NAM. Each point represents one SNP. Additive effect values were converted to absolute values before analysis to evaluate the relationship between effect size magnitude and association strength, regardless of effect direction. Pearson’s correlation coefficient (*r*) and corresponding *p*-value were calculated separately for each population combination. The blue solid line represents the fitted linear regression trend line, and the gray shaded area indicates the 95% confidence interval around the regression line. **A-H**, Scatter plots showing the relationship between the statistical significance (−log_10_(*p*)), additive effect values, and allele frequency of 20 SNPs identified in the whole-population GWAS (n = 168). **I-P**, Scatter plots showing the relationships between association strength (−log_10_(*p*)), additive effect values, and allele frequency for 13 SNPs identified in the GWAS of the other three population combinations.
**Supplemental Figure S9.** Manhattan plots and Q-Q plots of GWAS analyses for the RDA (n = 93) and RDA + CNU combinations (n = 142) for three traits. **A-F**, Manhattan plots of the three traits. Intervals colored red in the middle of the Manhattan plot represent the ranges of kernel-related QTLs on chromosomes. Text labels of nearby colored intervals and SNPs represent their names reported in previous publications and identified in this study, respectively. The red and blue dashed lines indicate the suggestive (5.73) and simpleM significance threshold (6.52) respectively. The SNP *Chr7_144130795* was not identified only when NAM founders were included. **G-L**, Q-Q plot of each trait and combination.
**Supplemental Figure S10.** LD block and SNP genotype-based phenotype comparison of *Chr7_144130795*. **A**, Association mapping and pairwise LD analysis of genomic regions around *Chr7_144130795*. The red and blue dashed lines indicate the suggestive (5.73) and simpleM significance threshold (6.52) respectively. Red and blue dots represent the positions of SNPs exceeding the suggestive threshold and SNPs located nearby, respectively. Rectangular bars, marked with multiple colors and lines, highlight specific points corresponding to SNPs (pink), intergenic regions (orange), introns (light blue), and exons (dark blue) within the genomic regions. Black triangles in the lower panel highlight SNPs that exhibit high LD with the lead variant. The candidate gene, *Zm00001eb319930*, encodes a peptidyl-prolyl cis–trans isomerase (PPIase) (Table S5). **B**, Genotypes of each variant and their phenotypic distributions on eight kernel-related traits. Bars represent the mean, and error bars indicate the standard error (SE). Statistical significance is indicated by asterisks: *, p < 0.05; **, p < 0.01; and ***, p < 0.001. While *Chr7_144130795* was associated with AS, PR, and kernel length, single-SNP genotype analysis revealed significant differences in kernel width, which showed clear differences despite the lack of statistical significance for this SNP in GWAS results.

**Supplemental Table S1.** Biosample ID list of 168 maize accessions used in the previous and the present studies.

**Supplemental Table S2.** Thresholds determined by different correction methods across four population combinations and traits.
**Supplemental Table S3.** Descriptive statistics of the 168 maize inbred lines in each subpopulation.
**Supplemental Table S4.** Descriptive statistics of the 168 maize inbred lines across kernel types.
**Supplemental Table S5.** Candidate genes located around the 33 SNPs identified across four population combinations.
**Supplemental Table S6.** Statistical significance (−log_10_(*p*)) of the 33 SNPs identified by GWAS across four population combinations.
**Supplemental Table S7.** Allele frequencies of the 33 SNPs identified by GWAS across four population combinations.
**Supplemental Table S8.** Additive effects of the 33 identified SNPs across four population combinations in the MLM. **Supplemental Table S9.** Previously reported maize kernel-related QTLs and overlapping or nearby SNPs identified in this study.
